# Neural correlates of emotional reactivity and regulation in traumatized North Korean refugees

**DOI:** 10.1038/s41398-021-01579-1

**Published:** 2021-09-03

**Authors:** Kyung Hwa Lee, Ha Young Lee, Inkyung Park, Yu Jin Lee, Nambeom Kim, Sehyun Jeon, Soohyun Kim, Jeong Eun Jeon, Seog Ju Kim

**Affiliations:** 1 Department of Psychiatry and Center for Sleep and Chronobiology, Seoul National University, College of Medicine and Hospital, Seoul, Republic of Korea; 2 Division of Child and Adolescent Psychiatry, Department of Psychiatry, Seoul National University Hospital, Seoul, Republic of Korea; 3 Department of Psychological and Brain Sciences, University of Iowa, Iowa City, Iowa USA; 4 Neuroscience Research Institute, Gachon University, Incheon, Republic of Korea; 5 Department of Psychiatry, Korea University Anam Hospital, Seoul, Republic of Korea; 6 Department of Neurology, Gangneung Asan Hospital, Gangwon-do, Republic of Korea; 7 Department of Psychiatry, Sungkyunkwan University College of Medicine, Samsung Medical Center, Seoul, Republic of Korea

**Keywords:** Human behaviour, Scientific community

## Abstract

Refugees often report heightened emotional reactivity and emotion regulation difficulties and are at high risk for mental health problems. Given that refugees are repeatedly exposed to traumatic events that may cause changes in the brain, the present study examined neural correlates of emotional reactivity and regulation and their associations with refugee features (e.g., cumulative trauma) and the severity of psychiatric symptoms (e.g., post-traumatic stress disorder [PTSD]) in North Korean (NK) refugees. Forty NK refugees with trauma exposure and varying levels of psychopathology and 41 healthy South Korean (SK) controls without trauma exposure participated in this study. They performed an emotion regulation task during a functional magnetic resonance imaging (fMRI) assessment. Region of interest (ROI), whole brain, and generalized psychophysiological interaction (gPPI) analyses were conducted. NK refugees with trauma exposure and varying levels of psychopathology showed increased activation in response to negative socio-affective pictures in regions involved in affective processing, including the amygdala and hippocampus, relative to healthy SK controls without trauma exposure. They also exhibited greater prefrontal cortex (PFC) activation, amygdala–PFC functional connectivity (FC), and hippocampal–PFC FC during emotion regulation. More severe PTSD symptoms were associated with greater hippocampal response to negative pictures (vs. neutral pictures) in NK refugees. This study provides neuroscientific evidence for neural alterations in association with emotional reactivity and regulation in traumatized refugees. These findings may contribute to a better mechanistic understanding of emotional reactivity and regulation in refugees and suggest potential ways to address the emotional and mental problems of traumatized refugees.

## Introduction

Refugee resettlement and adjustment to a new society or country have been recognized as important issues, given the continued increase in the number of refugees worldwide [1]. Mental health problems may be the most critical issue; refugees are known to be at high risk for various mental health problems, such as depression, anxiety, and post-traumatic stress disorder (PTSD) [2, 3]. This may be related to the fact that refugees have repeatedly experienced stressful and traumatic events such as persecution, abuse, and violence during residency in their home country and their escape to or resettlement in different countries [4, 5]. Mental health problems in traumatized refugees may also hinder successful resettlement [6]. Research focusing on elucidating potential risks and maintaining factors in refugees’ mental health problems may contribute to promoting refugees’ successful adjustment to new countries.

One important factor contributing to mental health problems in traumatized refugees may be altered emotion processing, such as (1) heightened emotional responses to past or current traumatic events (emotional reactivity) and (2) limited ability to manage such heightened negative emotions (emotion regulation). It is essential to understand how refugees process negative emotional information and how they regulate their emotions. There is some evidence that refugees exhibit increased negative emotional responses to pictures depicting trauma-related scenes, such as interpersonal violence, where such images may be particularly relevant to refugees [7, 8]. Previous studies have also demonstrated that refugees who have had traumatic experiences report difficulties in regulating their emotions [9, 10] and lower emotion regulation capacity [11]. Furthermore, heightened emotional reactivity and emotion regulation difficulties in refugees vary according to the severity of their mental health problems including PTSD and depressive symptoms [9–12].

However, most previous studies used subjective measures (e.g., self-report ratings and questionnaires) to assess emotional responses to trauma-related stimuli and emotion regulation difficulties. These subjective methods are known to be biased [13]. Assessing emotion processing using such subjective measures may be especially problematic for refugees, who have difficulties in identifying and describing their own emotions [14, 15]. It has been suggested that neurobiological measures of emotion may improve our understanding of how people respond to emotional stimuli and regulate negative emotions [16, 17]. Furthermore, traumatic experiences affect brain function and connectivity as it pertains to a wide range of processes, including stress and emotional processing [18, 19]. Thus, it is important to examine whether traumatized refugees show altered neural correlates of emotional reactivity and regulation.

Neuroimaging studies have elucidated the neural circuits involved in emotional reactivity and regulation. Increased emotional responses to negative stimuli (emotional reactivity) are associated with greater activation in brain regions involved in affective processing, including the amygdala, hippocampus, and insula [20]. To date, only one functional magnetic resonance imaging (fMRI) study has demonstrated neural responses to negative faces in traumatized refugees [21]. Cumulative trauma of refugees was correlated with increased activity in response to fearful faces in the insula and anterior cingulate cortex (ACC) regions and decreased functional connectivity (FC) between the anterior insula and inferior frontal gyrus while looking at fearful faces. This result indicates altered emotional reactivity to fearful stimuli in refugees. Other fMRI studies demonstrated that individuals who experienced traumatic events or who had PTSD showed increased amygdala and hippocampal activity in response to negative pictures [22, 23].

In contrast to neural correlates of emotion reactivity, emotion regulation often appears to be associated with activation in prefrontal cortex (PFC) regions involved in cognitive control, including the dorsolateral prefrontal cortex (DLPFC), ventral lateral prefrontal cortex (VLPFC), and medial prefrontal cortex (MPFC) [24–26]. Emotion regulation has also been investigated in studies of subcortical–PFC FC [27]. There is no past fMRI research that examined neural correlates of emotion regulation in traumatized refugees. However, previous fMRI studies investigating neural correlates of emotion regulation in traumatized individuals with PTSD or without PTSD provided some potential alteration, such as reduced recruitment of PFC regions, during emotion regulation to decrease negative emotions [28, 29].

Taken together, these previous studies of traumatized individuals suggest that traumatized refugees may show increased neural response to negative information in the amygdala and hippocampus while they may show reduced recruitment of prefrontal regions during emotion regulation. However, no neuroimaging research investigated neural correlates of emotional reactivity and emotion regulation in traumatized refugee sample. It is also plausible that traumatized refugees may differ from other traumatized individuals in part because they may suffer from various ongoing post-migration stressors, such as discrimination, lack of social support, and unemployment [30, 31]. For these reasons, it is necessary to examine neural activation while traumatized refugees were performing an emotion regulation task where they were instructed to view trauma-related pictures (e.g., pictures depicting physical assaults relevant to refugees) and to voluntarily regulate emotional responses elicited by the pictures. Using the emotion regulation task and refugees’ trauma-related pictures may allow us to elucidate neural correlate of emotional reactivity and regulation in traumatized refugees.

In this study, we aimed to examine neural correlates of emotional reactivity and emotion regulation in traumatized refugees. To accomplish our goal, we recruited traumatized North Korean (NK) refugees who have settled in South Korea after escaping from North Korea. NK refugees were known to experience various types of stressful and traumatic events, including persecution, abuse, and violence during residency in North Korea and their journeys from North Korea to South Korea. South Korean (SK) adults were recruited as a control group without trauma and any psychiatric disorders. NK refugees and SK controls were scanned while they were performing the emotion regulation task. Of particular interest, we used emotion suppression as an emotion regulation strategy. One reason for this was that individuals who had alexithymic tendencies or PTSD symptoms were more likely to use emotion suppression [32, 33], suggesting that NK refugees may be more likely to use emotion suppression. Emotion suppression also appeared to activate PFC regions including the lateral prefrontal cortex (LPFC) [34, 35]. Given the paucity of evidence about neural correlates of emotion reactivity and regulation in traumatized refugee sample, we developed our hypotheses based on the results from previous imaging studies of traumatized individuals [22, 28]. We hypothesized that, relative to SK controls, NK refugees would show greater neural activation in response to negative pictures in subcortical–limbic regions (e.g., amygdala and hippocampus) while they would show less activation in the PFC (e.g., LPFC and MPFC) and lower subcortical–PFC connectivity during emotion regulation.

Furthermore, we attempted to examine whether the degree of depression, anxiety, and PTSD severity as well as refugee features (i.e., cumulative trauma and length of SK residency) were correlated with altered neural activation of emotional reactivity and regulation in NK refugees. Given the associations between altered emotion processing, cumulative trauma, and clinical features (i.e., psychiatric symptoms) [10, 21], we predicted that refugee features and the severity of depression, anxiety, and PTSD symptoms in NK refugees would be associated with neural activation in regions involved in emotional reactivity and regulation.

## Methods

### Participants

Ninety-three adults, including 49 NK refugees and 44 SK controls, were initially recruited through advertisements from 2013 to 2017 [15, 36]. SK who had not been exposed to trauma were recruited as healthy controls; 12 participants were excluded due to anatomical abnormalities (e.g., tumor) (*n* = 4 [4 NK refugees]), task-related errors (*n* = 6 [4 NK refugees and 2 SK controls]), or poor image quality due to excessive head motion (*n* = 2 [1 NK refugee and 1 SK control], see the Supplement for the exclusion criteria of excessive head motion). Thus, our final sample comprised 40 NK refugees (31 females; mean ± SD age, 36.15 ± 10.94 years) and 41 SK controls (28 females; mean ± SD age, 36.54 ± 11.45 years). Given that a sample size of 40 was known to be adequate to identify regions with large effect sizes (Cohen’s *d* > 0.8) in task-based fMRI group analyses [37], our sample size might be sufficient to detect large effect sizes. Participants were excluded if they had (a) any metal or other implants that contravened MRI safety standards and/or (b) a history of head injury, neurological disorder, untreated serious medical illness, and/or a neurodevelopmental disorder. SK controls were excluded if they had a lifetime history of a psychiatric disorder.

### Procedures

This study was approved by the Institutional Review Board of Seoul National University Hospital. All participants provided written informed consent before participating in the study. The participants visited our center on two occasions. During the first visit, both NK and SK participants were assessed using the Structured Clinical Interview for the Diagnostic and Statistical Manual of Mental Disorders, Fourth Edition (DSM-IV) [38] and were asked to complete questionnaires assessing their clinical characteristics. NK refugees also completed the Clinician-Administered PTSD Scale-IV (CAPS-IV) [39] and a short interview after the clinical assessment, in which they were asked to briefly describe their life history and types of traumatic experiences. During the second visit, they participated in an fMRI assessment while performing an emotion regulation task. Participants were given the task instructions and practiced the task prior to the fMRI assessment.

### Clinical assessments and self-report measures

The CAPS-IV is a 30-item structured interview concerned with current and lifetime diagnoses of PTSD based on the DSM-IV. The current and lifetime CAPS-IV scores used in the present study were the sums of the current and lifetime symptom severity scores, respectively. The Beck Depression Inventory is widely used for the assessment of depressive symptom severity during the past 2 weeks. It is a self-report scale with 21 items assessing various symptoms of depression such as cognitive, emotional, physical, and motivational symptoms using a 4-point Likert scale. The Korean version has been validated and demonstrated an excellent internal consistency [13, 40]. The Beck Anxiety Inventory (BAI) is a 21-item self-report questionnaire that assesses common symptoms of anxiety during the past week using a 4-point Likert scale. In the present study, the Korean version of the BAI, previously validated [41], was used. The revised Toronto Alexithymia Scale (TAS) is a 23-item self-report questionnaire that measures alexithymia based on three subscales, including difficulty describing feelings, difficulty identifying feelings, and externally oriented thinking. This study used the Korean version of the TAS, which has shown good internal consistency [42]. The Trauma Exposure Check List for NK Refugees was used to explore the types of traumatic events and count the number of traumatic experiences [43].

### fMRI emotion regulation task

As in a previous study [28], each trial began with the presentation of a fixation cross for 1 s followed by negative socio-affective pictures or neutral pictures (4 s). An emotion regulation cue was superimposed on the center of the picture for 1 s, and the picture continued to be displayed while emotions were regulated (i.e., suppressed or maintained) for the next 7 s. Then participants were asked to rate the intensity of their emotions based on a rating scale (1 = neutral, 2 = negative, 3 = very negative) presented for 4 s, which was followed by a fixation dot (4 s). In the “suppress” condition, participants were instructed to suppress their emotional response to negative socio-affective pictures (i.e., try not to feel any emotions). In the “maintain” condition, they were asked to maintain their responses to negative socio-affective and neutral pictures.

In total, 36 negative socio-affective pictures (18 pictures per each emotion regulation condition) and 18 neutral pictures (only for the “maintain” condition) were used. Neutral pictures were not used for the “suppress” condition. These negative and neutral pictures, which were taken from Korean Social Affective Visual Stimuli [44], have been validated. For example, negative stimuli induced negatively valenced emotions and neutral stimuli were rated as neutral, respectively. Negative pictures depicted negatively valenced social situations such as a person suffering from physical abuse by other people and neutral pictures included neutral social situations such as people walking together.

### fMRI data acquisition and analysis

The fMRI data were acquired with a 3 T whole-body Tim Trio scanner (Siemens AG, Erlangen, Germany) using a 12-channel birdcage head coil and interleaved T2*-weighted echo planar imaging sequence. High-resolution structural images were acquired with a T1-weighted 3D gradient echo pulse sequence with magnetization-prepared rapid gradient-echo sequencing. The fMRI data were preprocessed and analyzed using SPM12 (Wellcome Trust Centre for Neuroimaging, London, UK). Both regions of interest (ROIs) (priori-defined regions; Fig. S1) and exploratory whole-brain *t* tests were used to elucidate brain regions showing group differences in emotional reactivity (“looking at negative pictures” vs. “looking at neutral pictures” contrast) and emotion regulation (“suppressing emotions” vs. “looking at negative pictures” contrast). A generalized psychophysiological interaction (gPPI) analysis [45] using the CONN connectivity toolbox in SPM 12 [46] was performed to examine task-dependent FC between the seed regions (i.e., amygdala and hippocampus) and other brain regions. Further information on the fMRI data acquisition and analysis is described in the Supplement.

### Statistical analysis

Statistical analyses were conducted using the SPSS 25.0 software (SPSS Inc., Chicago, IL, USA). Independent-sample *t* tests were performed to test for group differences in demographic characteristics, clinical features, and neural activation (e.g., mean parameter estimates of emotional reactivity [“looking at negative pictures” vs. “looking at neutral pictures” contrast] and emotion regulation [“suppressing emotions” vs. “looking at negative pictures” contrast]) extracted from the ROIs involved in emotional reactivity and regulation. Chi-squared tests were used for group differences in categorical variables. Repeated-measures analyses of variance (ANOVAs) were used to examine whether group differences in behavioral ratings were affected by the emotion regulation conditions. Correlation analyses were conducted to explore whether refugee features (e.g., the number of traumatic experiences) and clinical features (e.g., psychiatric symptoms) were associated with mean neural activation (e.g., mean parameter estimates of the contrasts) and mean FC extracted from the anatomically and functionally defined ROIs in NK refugees. The Benjamini–Hochberg method with a false discovery rate (FDR) of 0.05 was applied to correct for multiple correlation tests [47].

## Results

### Demographic and clinical features

The demographic and clinical features of NK refugees and SK controls are presented in Table 1. No significant differences in age or gender were observed between NK refugees and SK controls. NK refugees reported more severe depressive symptoms and anxiety and more difficulty in identifying feelings than SK controls.

**Table 1:** Demographic and clinical characteristics of the study participants.

Variables	South Korean (SK) controls (*n* = 41)	North Korean (NK) refugees (*n* = 40)	Group comparison	
	Mean (SD)	Mean (SD)	*t* or *x*^2^	Sig. (two-tailed)
Age (years)	36.54 (11.45)	36.15 (10.94)	0.16	0.88
Females, *n* (%)	28 (68.3)	31 (77.5%)	*x*^2^ = 0.87	0.35
Refugee features				
Time since settlement in South Korea (years)	–	5.57 (2.79)	–	–
Number of traumas experienced (*n*)	–	4.58 (2.96)	–	–
Axis-I psychiatric disorder, *n* (%)				
Post-traumatic stress disorder	–	5 (20%)	–	–
Mood disorder	–	7 (17.5%)	–	–
Eating disorder	–	1 (2.5%)	–	–
Generalized anxiety disorder	–	1 (2.5%)	–	–
Psychotropic medication				
Anti-anxiety and hypnotics	–	2 (5%)	–	–
Anti-depressant	–	1 (2.5%)	–	–
Current CAPS-IV Total	–	20.95 (22.38)	–	–
Lifetime CAPS-IV Total	–	39.25 (31.41)	–	–
BDI^a^	8.95 (8.58)	15.69 (14.26)	2.49	0.015
BAI^b^	6.39 (7.23)	19.59 (14.43)	4.95	<0.001
TAS^c^				
TAS total	27.45 (11.44)	32.84 (15.32)	1.43	0.16
TAS—DIF	4.72 (5.05)	10.18 (7.36)	3.37	<.01
TAS—DDF	8.68 (4.64)	8.95 (4.71)	0.21	0.83
TAS—EOT	14.05 (3.64)	13.72 (5.40)	−0.25	0.80

There are some missing data on self-report measures (^a^39 SK vs. 35 NK; ^b^38 SK vs. 32 NK; ^c^22 SK vs. 37 NK).

*CAPS-IV* Clinical-administered PTSD scale-IV, *BDI* Beck Depression Inventory, *BAI* Beck Anxiety Inventory, *TAS* Toronto Alexithymia Scale, *TAS—DIF* TAS—Difficulty Identifying Feeling, *TAS—DDF* TAS—Difficulty Describing Feelings, *TAS—EOT* TAS—Externally Oriented Thinking.

### Behavioral ratings

As in previous study [48], we conducted two repeated-measures ANOVAs on ratings after emotion regulation conditions.

#### Responses to negative pictures vs. neutral pictures

A group (NK vs. SK) × condition (maintaining responses to negative vs. neutral pictures) repeated-measures ANOVA revealed a significant main effect of the condition (*F*[1, 74] = 1296.29, *p* < 0.001, partial *η*^*2*^ = 0.95), indicating that both NK refugees and SK controls reported more negative emotional responses after maintaining responses to negative pictures compared to neural pictures (Fig. S2-a).

#### Suppressing vs. maintaining responses to negative pictures

A group (NK vs. SK) × condition (suppressing vs. maintaining responses to negative pictures) repeated-measures ANOVA revealed a significant group × condition interaction effect (*F*[1,74) = 4.71, *p* < 0.03, partial *η*^2^ = 0.06) (Fig. S2-b). SK controls reported less intense emotional responses after suppressing compared to maintaining responses to negative pictures (*t*[39] = 3.32, *p* = 0.002, Cohen’s *d* = 0.52), but NK refugees reported similar emotional responses regardless of the emotion regulation conditions (*t*[35] = 0.69, *p* = 0.50). This result indicated that NK refugees had more difficulty in suppressing negative emotions or were less successfully suppressing emotions than SK controls.

### Neural activation in response to negative pictures (emotional reactivity)

#### ROI analysis

**Fig. 1:**
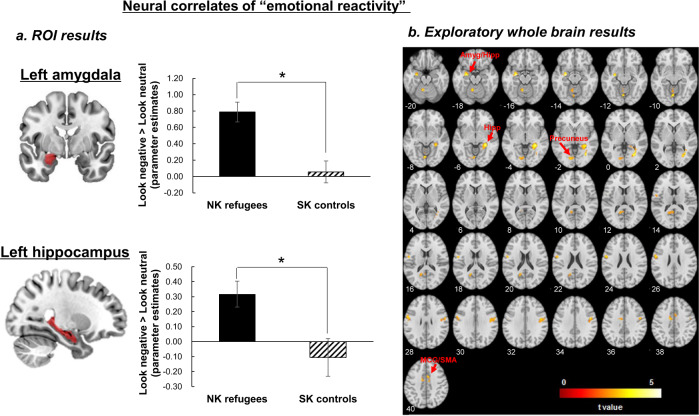
Neural correlates of emotional reactivity. **a** Neural activation in response to negative socio-affective pictures (vs. neutral pictures) in the anatomically defined amygdala and hippocampus ROIs between NK refugees and SK controls (*p < 0.05), **b** Results from the exploratory whole-brain analysis showing significant group differences in neural activation in response to negative socio-affective pictures (vs. neutral pictures) between NK refugees and SK controls (cluster-defining threshold, *p* < 0.001; cluster size >80 voxels to achieve a cluster-wise corrected *p* < 0.05). *ROI* region of interest, *NK* North Korean, *SK* South Korean, *Amyg/Hipp* amygdala extending to hippocampus, *Hipp* hippocampus, *MCG* middle cingulate gyrus, *SMA* supplementary motor area.

Consistent with our hypothesis, NK refugees exhibited greater activation in the left amygdala (*t*[79] = 4.09, *p* = 0.0001, Cohen’s *d* = 0.91) and bilateral hippocampus (left: *t*[79] = 2.75, *p* = 0.007, Cohen’s *d* = 0.61; right: *t*[79] = 3.38, *p* = 0.001, Cohen’s *d* = 0.75) (Fig. 1a), compared to SK controls, but not in the right amygdala or bilateral anterior insula (all *p*s > 0.07) in response to negative socio-affective pictures compared to neutral pictures.

#### Exploratory whole-brain analysis

Similar to the ROI results, the whole-brain analysis revealed more amygdala and hippocampal activation in NK refugees compared to SK controls in response to negative socio-affective pictures vs. neutral pictures (Table S1 and Fig. 1b; cluster-defining threshold, *p* < 0.001; cluster size >80 voxels to achieve a cluster-wise corrected *p* < 0.05). NK refugees also showed greater activation in response to negative vs. neutral pictures in other cortical regions, including the cingulate cortex, precuneus, and visual cortex, than SK controls. However, SK controls did not show greater activation in any regions compared to NK refugees in response to negative vs. neutral pictures.

### Neural activation and FC during emotion regulation

#### ROI analysis

**Fig. 2:**
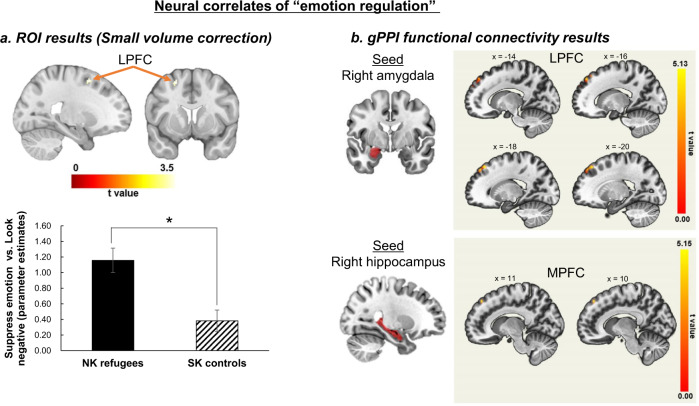
Neural correlates of emotion regulation. **a** Left lateral prefrontal cortical (LPFC) activation during emotion suppression (vs. looking at negative pictures) resulted from the small volume correction (SVC) analysis limited to anatomically defined prefrontal ROI masks between NK refugees and SK controls (cluster-defining threshold, *p* < 0.001; cluster size >25 voxels to achieve a SVC corrected *p* < 0.05) (*p < 0.05). **b** Increased functional connectivity (1) between right amygdala (seed) and left LPFC (center coordinate: *x* = 18, *y* = 36, *z* = 52) and (2) between right hippocampus (seed) and MPFC (center coordinate: *x* = 10, *y* = 44, *z* = 48) during emotion suppression (vs. looking at negative pictures) in NK refugees compared to SK controls (FWE corrected threshold of *p* < 0.05). *ROI* region of interest, *NK* North Korean, *SK* South Korean, *gPPI* generalized psychophysiological interaction.

No significant differences in activation were observed during emotion suppression (vs. looking at negative pictures) between NK refugees and SK controls in the DLPFC, VLPFC, and MPFC ROI masks (all *p*s > 0.40). However, the small volume correction (SVC) analysis limited to the prefrontal ROI revealed a cluster of activation in the left LPFC (cluster-defining threshold, *p* < 0.001; cluster size >25 voxels to achieve a SVC corrected *p* < 0.05). In this LPFC cluster (*xyz* coordinate = −22, 8, 56; 31 voxels; peak *t* value = 3.73), NK refugees showed greater activation than SK controls during emotion regulation (vs. looking at negative pictures; mean activation in the LPFC cluster, *t*[79] = 3.71, *p* = 0.0001, Cohen’s *d* = 0.82) (Fig. 2a).

#### Exploratory whole-brain analysis

NK refugees showed more activation during emotion regulation (vs. looking at negative pictures) in prefrontal regions, in addition to several other regions, compared to the SK controls (cluster-defining threshold, *p* = 0.001), but the differences were not significant after correcting for multiple comparisons (cluster-defining threshold, *p* < 0.001; cluster size >89 voxels to achieve a cluster-wise corrected *p* < 0.05). These exploratory whole-brain results are shown at an uncorrected *p* < 0.001, with a minimal cluster size of 10 (Table S2 and Fig. S3).

#### FC using gPPI

NK refugees had greater FC between the right amygdala and LPFC and between the right hippocampus and dorsomedial prefrontal cortex during emotion suppression (vs. looking at negative pictures) compared to SK controls (Fig. 2b and Table 2). However, no significant group differences in FC were observed between the prefrontal and other seed regions (i.e., left amygdala and left hippocampus).

**Table 2:** Functional connectivity between subcortical and prefrontal regions using the amygdala and hippocampus seeds during emotion suppression (vs. looking at the negative pictures).

Seed	Contrast	Cluster	Region	BA	H	Number of voxels in the region	Cluster size (voxels)	MNI coordinates			Peak *t*
								*x*	*y*	*z*	
R amygdala	NK > SK	1	Superior frontal gyrus	8	L	205	293	−18	36	52	5.13
			Medial frontal gyrus	10		71					
			Middle frontal gyrus	9		4					
		2	Middle frontal gyrus	8	L	70	82	−32	16	50	4.16
			Precentral gyrus			12					
	SK > NK		—								
L amygdala	NK > SK	1	Cerebellum		R	77	77	54	−52	−46	4.55
	SK > NK		—								
R hippocampus	NK > SK	1	Medial frontal gyrus	8	L	46	103	10	44	48	5.15
			Medial frontal gyrus	8	R	44					
			Superior frontal gyrus			10					
		2	Angular gyrus	39	L	78	78	−46	−64	32	4.25
	SK > NK	1	Rectus gyrus		R	37	95	12	20	−16	4.6
			Olfactory gyrus			29					
			Superior orbital gyrus	47		14					
			Inferior orbital gyrus	47		7					
			Medial orbital gyrus	47		2					
L hippocampus	NK > SK		—								
	SK > NK		—								

*BA* Brodmann area, *H* hemisphere, *NK* North Korean refugees, *SK* South Korean controls, *R* right, *L* left.

### Correlations between refugee features, clinical features, neural activation, and FC in NK refugees

#### Correlations between refugee features, neural activation, and FC

As shown in Table 1, two refugee features, including “length of SK residency (i.e., time since settlement in SK)” and “cumulative trauma (i.e., number of traumatic experiences),” were used in these analyses. Given the wide age range and gender disparity, age and gender were included as covariates. There were significant correlations between cumulative trauma and PTSD symptoms (i.e., traumatic experiences and PTSD [lifetime CAPS-IV: *r* = 0.50, *p* < 0.01; current CAPS-IV: *r* = 0.60, *p* < 0.001]). Current and lifetime PTSD symptoms were further controlled to test correlation between cumulative trauma, neural activation, and FC. Cumulative trauma was not significantly correlated with neural activation in regions involved in emotional reactivity and emotion regulation and FC between subcortical–PFC regions in NK refugees (all *p*s > 0.08). Length of SK residency was not significantly correlated with any clinical, neural activation, and FC (all *p*s > 0.08).

#### Correlations between clinical features, neural activation, and FC

Given the wide age range, gender disparity, and close relationships among variables in NK refugees (i.e., traumatic experiences and PTSD symptoms, alexithymia and depression [*r* = 0.44, *p* < 0.05] and alexithymia and anxiety [*r* = 0.50, *p* < 0.01]), all correlation coefficients were computed controlling for age, gender, the number of traumatic experiences, and alexithymia scores. Depressive symptoms were highly correlated with anxiety symptoms (*r* = 0.51, *p* < 0.01) but not with current PTSD symptoms (*r* = 0.33, *p* = 0.09) and lifetime PTSD symptoms (*r* = 0.35, *p* = 0.07) after controlling for age, gender, the number of traumatic experiences, and alexithymia scores.

**Fig. 3:**
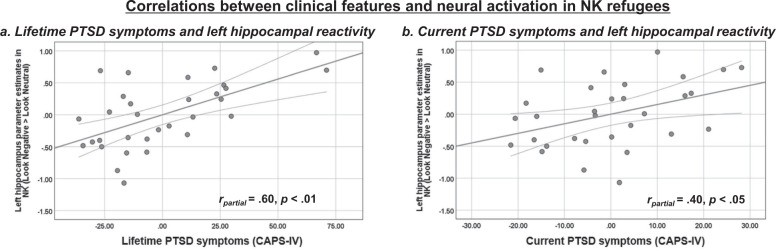
Partial correlations between clinical features and neural activation (emotion reactivity) within NK refugees, controlling for age, gender, the number of traumas, and alexithymia. **a** Partial correlation between lifetime PTSD symptoms and left hippocampal activity in response to negative socio-affective pictures (vs. neutral pictures), **b** Partial correlation between current PTSD symptoms and left hippocampal activity in response to negative socio-affective pictures (vs. neutral pictures) (FDR corrected *p* < 0.05). *NK* North Korean, *PTSD* post-traumatic stress disorder, *CAPS-IV* Clinician-Administered PTSD Scale-IV.

NK refugees who had greater lifetime PTSD symptoms exhibited greater activity in response to negative (vs. neutral) pictures in both the left (*r* = 0.60, *p* < 0.01) (Fig. 3a) and right hippocampus (*r* = 0.51, *p* < 0.01). NK refugees who had current PTSD also showed greater left hippocampal activity in response to negative (vs. neutral) pictures (*r* = 0.40, *p* < 0.05) (Fig. 3b). NK refugees who had more severe depressive symptoms showed greater amygdala activity in response to negative (vs. neutral) pictures (*r* = 0.49, *p* < 0.05), but this result did not remain significant after correction for multiple correlation tests (Fig. S4a). The anxiety scores of NK refugees were not correlated with neural activation in response to the negative pictures (all *ps* > 0.06).

More severe lifetime PTSD symptoms were significantly associated with lower FC between the right amygdala and LPFC in NK refugees during emotion suppression (*r* = −0.41, *p* < 0.05), but this finding did not remain significant after correction for multiple correlation tests (Fig. S4b). Other clinical features were not significantly correlated with prefrontal activation and FC in NK refugees during emotion suppression (all *p*s > 0.07).

### Sensitivity analysis

Given that there were 14 NK refugees with Axis-I psychiatric disorders, it is possible that our findings may be driven by current psychiatric disorders. To rule out this possibility, we investigated group differences in neural activation and FC between NK refugees without current Axis-I psychiatric disorders and NK refugees with current Axis-I psychiatric disorders. Given significant differences in age and the number of traumatic experiences between these two groups, we controlled age and the number of traumatic experiences. We found no significant group differences, indicating that our main findings were not affected by current Axis-I psychiatric disorders in NK refugees.

## Discussion

In the present study, NK refugees with trauma exposure and varying levels of psychopathology showed greater amygdala and hippocampal responses to negative socio-affective pictures compared to healthy SK controls without trauma exposure. NK refugees also exhibited greater PFC activation, amygdala–PFC FC, and hippocampal–PFC FC during emotion regulation compared to SK controls. Furthermore, more severe PTSD symptoms were significantly correlated with greater hippocampal activity in response to negative pictures in NK refugees. However, refugee features such as cumulative trauma and length of SK residence were not related to neural activation in regions of emotional reactivity and regulation in NK refugees.

### Neural correlates of emotional reactivity in NK refugees

Consistent with our first hypothesis, we found greater amygdala and hippocampal activity in response to negative pictures than neutral pictures in NK refugees compared to SK controls. These results were aligned with extant literature that showed heightened amygdala and hippocampal activity in response to trauma-related negative pictures in individuals who had traumatic experiences [22, 23]. The amygdala and hippocampus are involved in several emotion processes, including basic emotions (e.g., fear and anger), hypervigilance/arousal, emotional memory, and emotion-cognition interactions [49, 50]. Individuals who experienced stressful and traumatic events may be highly sensitive to negative information that may contribute to increasing neural activity in these subcortical regions. Heightened amygdala and hippocampal responses to negative pictures in NK refugees may represent increased vigilance, arousal, negative emotions, and emotional memory pertaining to trauma-related information. These results suggest that heightened amygdala and hippocampal reactivity to trauma-related information may be neurobiological factors contributing to mental health problems in traumatized refugees. Alleviating heightened emotional reactivity to trauma-related information may be key to improving mental health for traumatized refugees.

### Neural correlates of emotion regulation in NK refugees

Inconsistent with our second hypothesis, NK refugees showed greater PFC activation and subcortical–PFC (i.e., amygdala–PFC and hippocampus–PFC) FC during emotion regulation compared to SK controls. These findings were inconsistent with previous research demonstrating reduced PFC recruitment during emotion regulation in traumatized individuals [28, 29]. This may be due to using different emotion regulation strategies. Unlike previous studies using cognitive reappraisal, we used emotion suppression as an emotion regulation strategy. As mentioned earlier, alexithymic individuals or individuals with PTSD were more likely to use emotion suppression to decrease emotions. Based on the interference hypothesis [51], greater use of emotion suppression in traumatized individuals may interfere with using instructed cognitive reappraisal. Such interference may contribute to difficulties in recruiting PFC regions during cognitive reappraisal. However, in our study, traumatized refugees might be able to recruit PFC regions when they were asked to decrease emotion using suppression. Thus, NK refugees’ frequent, or even habitual, use of emotion suppression as a coping mechanism may be associated with greater PFC activation and subcortical–PFC connectivity. Similarly, NK refugees show strong amygdala–PFC connectivity even during the resting state [15], suggesting that they readily engage in emotion suppression when they feel negative emotions. However, future research is needed to examine whether traumatized refugees have difficulty in using cognitive reappraisal or recruiting PFC during cognitive reappraisal.

Despite their readily engaging in emotion suppression (e.g., greater resting-state amygdala–DLPFC FC) in the absence of externally presented stimuli or tasks [15], NK refugees may need to make more effort to decrease heightened negative emotions induced by negative pictures. In other words, heightened amygdala and hippocampal activity through bottom–up emotion generation using external stimuli may contribute to boosting prefrontal downregulation of negative emotions. Thus, greater PFC activation and subcortical–PFC connectivity during emotion suppression may reflect greater effort to regulate heightened negative emotions in NK refugees, who showed more intense responses to negative socio-affective pictures than SK controls. In support of this idea, previous research has shown that increased emotional responses to negative stimuli contribute to increased demands for regulation of emotional distress [52]. Children with high levels of violence exposure also showed greater subcortical (i.e., amygdala and hippocampus) and prefrontal cortical (i.e., ventromedial PFC) activation during response inhibition [53]. Similar to NK refugees with trauma exposure, children exposed to violence may make more effort (e.g., increased attention) to inhibit inappropriate responses, possibly due to increased emotional vigilance and memory associated with negative information (e.g., fearful faces). Although NK refugees made more effort to suppress emotions via enhanced PFC recruitment and subcortical–PFC connectivity, NK refugees failed to show reduction in subjective emotional responses (see behavioral ratings) to negative pictures after emotion suppression.

### Correlations between refugee features, clinical features, and neural correlates of emotional reactivity and regulation in NK refugees

Cumulative trauma, one of the refugee features, was significantly correlated with PTSD symptoms but not with anxiety and depressive symptoms. This result was in line with previous studies showing that individuals with the higher number of traumatic experiences reported greater PTSD symptoms [54]. Both refugee features (i.e., cumulative trauma and length of SK residency) were not significantly correlated with neural activation and FC associated with emotional reactivity and regulation. Specifically, this result was inconsistent with the previous study that showed the association between cumulative trauma and neural activation in response to fearful faces in the insula and perigenual ACC [21]. It is possible that cumulative trauma may be specifically associated with neural activation of fear or threat-related processing. Compared to the previous study that used only fearful faces, we used negative pictures that have been validated to induce various negative emotions including anger, disgust, and fear [44]. In this case, refugees may respond to non-specific negative emotions and activate the amygdala and hippocampus, which are known to be regions involved in various emotion processing, regardless of their cumulative traumatic experiences. Future research is needed to examine whether the association between cumulative trauma (or other refugee features) and neural activation is true for fear or threat-related processing or is generalized to other negative emotional processing.

As hypothesized, greater severity of PTSD symptoms was correlated with the enhanced hippocampal reactivity to negative pictures (vs. neutral pictures) in NK refugees. NK refugees with greater PTSD symptoms showed enhanced hippocampal activation that may be related to traumatic memories retrieved by negative pictures. This result was consistent with previous PTSD and trauma-related studies showing exaggerated hippocampal activity when remembering negative pictures and recalling negative autobiographical memory [55, 56]. This finding may suggest that interventional strategies should focus on desensitizing refugees with current or lifetime PTSD to traumatic emotional memories.

### Limitations

Several limitations of the present study should be discussed. First, we included only traumatized NK refugees, which limits the generalizability of our findings to other traumatized refugees. Thus, future research is needed to validate our findings in other traumatized refugee samples. Second, this study used emotion suppression as an emotion regulation strategy. Previous research showed that different emotion strategies recruit different brain regions [57]. Thus, although greater PFC activation and subcortical–PFC connectivity in NK refugees may be true in case of emotion suppression, further studies are needed on other emotion regulation strategies, such as cognitive reappraisal. Finally, we included healthy SKs as the control group, who share a similar heritage and history to NKs, as well as the same Korean language (with differences in vocabulary, accent, and so on), but were raised in distinct cultures and societies. Thus, NKs may differ from the SK control group in terms of several aspects, including different cultures, traumatic experiences, clinical features, and current stress levels (e.g., NKs’ greater stress due to discrimination, lack of social support, and financial problems). Ideally, a control group should be carefully selected according to the specific research goals. However, in practical terms, it is difficult to recruit NK refugees without traumatic experiences or SK with similar traumatic experiences (e.g., human trafficking and witnessing public executions). Alternatively, SK with any type of trauma exposure and some levels of psychopathology (e.g., PTSD symptoms) may be another good control group to disentangle the effect of being a refugee from the effects of trauma and psychopathology. It is also worthy to mention that the comparison of traumatized NK refugees with and without psychiatric disorders may be a good option to examine the effect of psychopathology in traumatized refugee sample. Surprisingly, there were only 5 NK refugees who were diagnosed PTSD and 9 NK refugees with other psychiatric disorders (i.e., 7 with mood disorders, 1 with eating disorder, and 1 with generalized anxiety disorder) at the time of inclusion of this study. However, we did not find significant differences in neural substrates between NK refugees with and without psychiatric disorders. Such null findings may be due in part to a relatively small sample size and heterogeneity in NK refugees with psychiatric disorders. Further research is needed to compare neural substrates between traumatized refugees with and without psychiatric disorders.

## Conclusions

Despite these limitations, this study had some strengths; for example, it is the first study to examine neural correlates of emotional reactivity and regulation in a traumatized refugee sample. Furthermore, the findings from this study have both clinical and methodological implications. Relative to healthy SK controls without trauma exposure, NK refugees with trauma exposure and varying levels of psychopathology may have heightened neural sensitivity to trauma-related information, which was varied by psychiatric symptoms, suggesting that future interventions should focus on alleviating such neural sensitivity and desensitizing refugees to traumatic emotional memories. With respect to emotion regulation, traumatized refugees may have the ability to regulate heightened negative emotions by recruiting PFC activation and subcortical–PFC FC, but they may need more effortful neural recruitment for emotion regulation. It is also possible that the emotion regulation ability of refugees may be underestimated or inappropriately assessed due to prejudice, stigma, cultural differences, or language barriers, such as differences in vocabulary and orthography. Refugees who suffer from mental health problems should be given special attention to improve their emotion regulation capabilities by encouraging use of more efficient emotion regulation and more appropriate emotion regulation strategies. Methodologically, given the refugees’ high levels of alexithymia and language problems, future research should include multiple assessments, including self-report, physiological, and neural measures, to evaluate emotion regulation difficulties in traumatized refugees subjectively and more objectively.

## Supplementary information

Supplementary information
