# Supplementary material for: Neural correlates of emotional reactivity and regulation in traumatized North Korean refugees
Source: Transl Psychiatry. 2021 Sep 3;11:452. doi: 10.1038/s41398-021-01579-1 (PMC8417257; doi:10.1038/s41398-021-01579-1)
Supplement: Supplementary file 1 — Supplementary information [file 41398_2021_1579_MOESM1_ESM.docx]

**Supplementary materials**

**Neural correlates of emotion reactivity and regulation in North Korean refugees**

Kyung Hwa Lee, PhD, Ha Young Lee, MA, Inkyung Park, MA, Yu Jin Lee, MD, PhD, Nambeom Kim, PhD, Sehyun Jeon, MD, Soohyun Kim, MA, Jeong Eun Jeon, MA,

Seog Ju Kim, MD, PhD

**Methods**

**fMRI data analysis**

***fMRI data acquisition and preprocessing.*** The fMRI data were acquired with a 3 T whole-body Tim Trio scanner (Siemens AG) using a 12-channel birdcage head coil and interleaved T2*-weighted echo planar imaging (repetition time [TR] = 2,000 ms, echo time [TE] = 30 ms, flip angle = 90°, slice thickness = 4.0 mm, in-plane resolution = 3.4 × 3.4 mm, no gap, 33 axial slices, field of view [FOV] = 220 mm, 220 volumes). High-resolution structural images were acquired with a T1-weighted 3D gradient echo pulse sequence with magnetization-prepared rapid gradient-echo sequencing (TR = 1670 ms, TE = 1.89 ms, flip angle = 9°, slice thickness = 1.0 mm, in-plane resolution = 1.0 × 1.0 mm, FOV = 250 mm).

The fMRI data were preprocessed using SPM12 (Wellcome Trust Centre for Neuroimaging, London, UK). The data were slice time-corrected, motion-corrected, co-registered with the high-resolution structural image, spatially normalized to Montreal Neurological Institute (MNI) space, and smoothed using a 6-mm full-width at half-maximum Gaussian kernel. Co-registered and normalized fMRI data were visually inspected for quality control. Artifact Detection Tools (ART; <http://www.nitrc.org/projects/artifact_detect/>) was used to identify outlier volumes. Outliers with significant head motion for each participant were detected if those were greater than 2 mm composite motion or had larger global mean intensity (i.e., difference in global mean intensity across functional volumes > 3 SD). The outlier volumes did not exceed 15% of the total volumes in any participants, so none were excluded from the final analysis. The outliers were also entered into the first-level general linear model (GLM) as nuisance regressors to remove possible artifacts.

***First-level analysis.*** First-level GLM analyses were conducted for each participant. Five regressors pertaining to the presentation of pictures (two regressors: “looking at negative pictures” and “looking at neutral pictures”) and the emotion regulation phase (three regressors: “suppressing emotion”, “maintaining emotion”, and “maintaining neutral”) were entered into the model. Other regressors were also created, corresponding to the fixation cross, rating phase, and fixation dot, but these were of no interest. Regressors were defined based on boxcar functions that were convolved with the canonical hemodynamic response function. Six head motion parameters and outliers were included in each participant’s GLM model, to control for the effects of head motion and outliers.

Based on previous research ^1^, contrast images were defined to identify brain regions showing greater activation when looking at negative pictures compared to neutral pictures (emotional reactivity: looking at negative pictures vs. looking at neutral pictures contrast) and when suppressing the emotional response to negative pictures compared to looking at negative pictures (emotion regulation: suppressing negative emotion vs. looking at negative pictures contrast). These contrast images were submitted to the second-level group analysis.

***Second-level group analysis.*** We first performed 1) ROI analyses using priori-defined regions. Our ROIs included three subcortical-limbic regions (brain regions associated with affective processing) known to be involved in emotional reactivity, i.e., the amygdala, hippocampus, and anterior insula, and three prefrontal areas known to be associated with emotion regulation, i.e., the DLPFC, VLPFC and MPFC (Figure S1). The ROIs were defined using automated anatomical labeling (AAL). We tested group differences in neural activation (emotional reactivity: looking at negative pictures > looking at neutral pictures) in subcortical-limbic regions and neural activation (emotion regulation: suppressing negative emotion > looking at negative pictures) in prefrontal regions using independent-sample *t*-tests in SPSS. Thus, we extracted mean parameter estimates from each subcortical-limbic ROI for the “looking at negative pictures > looking at neutral pictures” contrast, and from each prefrontal ROI for the “suppressing negative emotion > looking at negative pictures” contrast. Bonferroni correction was used to correct for multiple comparisons for several ROIs (i.e., for three bilateral subcortical-limbic regions related to emotional reactivity [*p* = 0.05/6 ROIs = 0.008], and two bilateral prefrontal regions and one medial prefrontal region relevant to emotion regulation [*p* = 0.05/5 ROIs = 0.01]). Given that our prefrontal ROIs were relatively large, we applied a small volume correction (SVC) to our prefrontal anatomical masks (using our prefrontal ROIs as an inclusive mask) and calculated the minimum cluster size required for an SVC-corrected *p* < .05 and cluster-defining threshold *p* < .001 (uncorrected) using 3dClustSim (version 18.3.16; AFNI; <https://afni.nimh.nih.gov>) ^2^. Thus, this SVC method helped us to examine prefrontal involvement during emotion regulation by identifying areas within prefrontal regions showing greater activation during suppressing negative emotions (vs. looking at negative pictures).

We conducted 2) whole brain analyses to determine regions showing group differences in neural activation, in association with emotional reactivity in the context of the “looking at negative pictures > look at neutral pictures” contrast, and in association with emotion regulation in the context of the “suppressing negative emotion > looking at negative pictures” contrast. Cluster-wise correction was performed in 3dClustSim, with smoothing estimated via AFNI’s 3dFWHMx with “acf” procedure (<https://afni.nimh,nih,gov>, version 18.3.16) ^2^, version 18.3.16). Cluster size was determined using 10,000 Monte Carlo simulations, second nearest neighbor (NN2) clustering, and a two-sided threshold. Both the cluster-defining threshold and cluster size necessary to achieve a cluster-wise corrected *p* < .05 are reported below.

To further examine group differences in subcortical-prefrontal FC during emotion regulation, we performed *3)* gPPI ^3^ using the connectivity toolbox CONN in SPM 12 ^4^. The deconvolved time-courses extracted from the seed regions including the amygdala and hippocampus were used as physiological regressors. The amygdala and hippocampus seed ROIs were derived from the Harvard-Oxford subcortical structural atlas ^5^ implemented in the CONN toolbox. The experimental conditions of emotion reactivity (e.g., looking at negative pictures) and emotion regulation (e.g., suppressing negative emotion) were used as psychological regressors. The interactions between the time-courses of our seeds and the experimental conditions were used as PPI regressors, which were convolved with the HRF. The CONN toolbox allowed us to create seed-to-voxel FC maps for each contrast (i.e., suppressing negative emotion vs. looking at negative pictures) and for each subject, and then to test group differences in FC. The results of the seed-to-voxel gPPI analysis were corrected for multiple comparisons at a family wise error rate (FWE) corrected threshold of *p* < .05.

**Statistical analysis**

***Correlations between clinical features and neural activation of emotional reactivity in NK refugees.*** To examine whether increased amygdala and hippocampal activities in response to negative pictures were associated with specific clinical characteristics of NK refugees, we performed correlation analyses including the clinical features of NK refugees (i.e., depression, anxiety, and PTSD symptoms) and extracted parameter estimates (looking at negative pictures > looking at neutral pictures contrast) from the anatomically defined amygdala and hippocampus ROIs, controlling for age, gender, the number of traumas, and alexithymia.

***Correlations between clinical features, neural activation, and functional connectivity during emotion regulation in NK refugees.*** Given the lack of any group differences in neural activation in the “suppressing negative emotion > looking at negative pictures” contrast for the anatomically defined prefrontal ROIs, we used parameter estimates extracted from the functionally defined prefrontal ROIs to explore associations between increased prefrontal activation during suppressing negative emotion (vs. looking at negative pictures) and clinical features (i.e., depression, anxiety, and PTSD symptoms) in NK refugees. Correlation coefficient estimates were extracted from regions showing significant FC with either the amygdala or hippocampus in the “suppressing emotion > looking at negative pictures” contrast, to test for correlations with clinical measures (i.e., depression, anxiety, and PTSD symptoms) in NK refugees. These analyses were similarly controlled for age, gender, the number of traumas, and alexithymia.

**Table S1**. Exploratory whole brain analysis to identify brain regions associated with emotion reactivity (“looking at negative pictures” vs. “looking at neutral pictures” contrast) (cluster-defining threshold, *p* < .001; cluster size > 80 voxels to achieve a cluster-wise corrected *p* < .05)

|  |  |  |  | # of voxels | Cluster size | MNI co-ordinates | | | Peak |
| --- | --- | --- | --- | --- | --- | --- | --- | --- | --- |
| Cluster | Region | BA | H | in region | (voxels) | *x* | *y* | *z* | T |
| *NK > SK in the “looking at negative pictures” vs. “looking at neutral pictures” contrast* | | | | | | | | | |
| 1 | Amgydala |  | L | 26 | 111 | -28 | -10 | -16 | 5.72 |
|  | Hippocampus | |  | 57 |  |  |  |  |  |
| 2 | Hippocampus | | R | 61 | 290 | 32 | -40 | -4 | 5.18 |
|  | Parahippocampal gyrus | 19 |  | 55 |  |  |  |  |  |
|  | Lingua gyrus | 19 |  | 42 |  |  |  |  |  |
| 3 | Cingulate gyrus | 24/32 | R | 209 | 495 | 4 | -8 | 48 | 4.91 |
|  | Supplement motor area | 6 |  | 253 |  |  |  |  |  |
| 4 | Lingua gyrus | 18 | L | 91 | 141 | -4 | -72 | -10 | 4.46 |
|  | Cerebellum |  |  | 25 |  |  |  |  |  |
|  | Vermis |  |  | 18 |  |  |  |  |  |
| 5 | Precentral gyrus | 6 | L | 76 | 132 | -54 | -2 | 16 | 4.31 |
|  | Postcentral gyrus | |  | 54 |  |  |  |  |  |
| 6 | Postcentral gyrus | 6 | R | 91 | 141 | 48 | -4 | 34 | 4.26 |
|  | Precentral gyrus | |  | 30 |  |  |  |  |  |
| 7 | Cerebellum |  | L | 76 | 87 | -10 | -54 | -20 | 4.18 |
| 8 | Precuneus | 30 | L | 64 | 113 | -14 | -54 | 22 | 3.81 |
|  | Cuneus |  |  | 22 |  |  |  |  |  |
|  | Calarine |  |  | 19 |  |  |  |  |  |
| *SK > NK in the “looking at negative pictures” vs. “looking at neutral pictures” contrast* | | | | | | | | | |
|  | No regions identified | |  |  |  |  |  |  |  |

Note. BA=Brodmann Area, H=Hemisphere, NK=North Korean refugees, SK=South Korean controls

**Table S2.** Exploratory whole brain analysis to identify brain regions associated with emotion regulation (“suppressing negative emotion” vs. “looking at negative pictures” contrast) (*p* < .001 uncorrected with a minimal cluster size of 10 voxels)

|  |  |  | |  | | # of voxels | Cluster size | | MNI coordinates | | | | Peak |
| --- | --- | --- | --- | --- | --- | --- | --- | --- | --- | --- | --- | --- | --- |
| Cluster | Region | BA | H | | | in region | | (voxels) | *x* | *y* | | *z* | T |
|  | *NK > SK in the “suppressing negative emotion” vs. “looking at negative pictures” contrast* | | | | | | | | | | | | |
| 1 | Anterior Insula | 13/47 | | L | | 21 | 21 | | -38 | | 12 | 4 | 4.17 |
| 2 | Superior frontal gyrus | 6 | | | R | 26 | 31 | | 20 | | 0 | 60 | 4.17 |
|  | Supplement motor area |  | |  | | 5 |  | |  | |  |  |  |
| 3 | Middle frontal gyrus | 6 | | L | | 31 | 61 | | -18 | | 10 | 60 | 3.97 |
|  | Superior frontal gyrus |  | |  | | 28 |  | |  | |  |  |  |
|  | Supplement motor area |  | |  | | 2 |  | |  | |  |  |  |
| 4 | Supramarginal gyrus | 40/2 | | L | | 24 | 25 | | -62 | | -24 | 30 | 3.89 |
|  | Post central gyrus |  | |  | | 1 |  | |  | |  |  |  |
| 5 | Fusiform gyrus | 19 | | L | | 11 | 24 | | -30 | | -60 | -4 | 3.78 |
|  | Lingual gyrus |  | |  | | 7 |  | |  | |  |  |  |
| 6 | Supplement motor area |  | | L | | 20 | 20 | | -8 | | 10 | 62 | 3.76 |
| 7 | Precuneus | 7 | | R | | 23 | 38 | | 20 | | -68 | 36 | 3.75 |
|  | Cuneus |  | |  | | 9 |  | |  | |  |  |  |
|  | Superior occipital gyrus |  | |  | | 6 |  | |  | |  |  |  |
| 8 | Superior frontal gyrus | 6/8 | | R | | 24 | 28 | | 24 | | 12 | 56 | 3.74 |
|  | Middle frontal gyrus |  | |  | | 4 |  | |  | |  |  |  |
| 9 | Precuneus | 7 | | R | | 9 | 10 | | 22 | | -52 | 44 | 3.71 |
| 10 | Anterior Insula | 13 | | R | | 60 | 63 | | 34 | | 20 | 8 | 3.63 |
|  | Inferior frontal gyrus | 47 | |  | | 1 |  | |  | |  |  |  |
| 11 | Superior parietal gyrus |  | | R | | 11 | 11 | | 28 | | -44 | 38 | 3.58 |
| 12 | Middle frontal gyrus | 8 | | R | | 11 | 14 | | 24 | | 36 | 32 | 3.55 |
|  | Superior frontal gyrus |  | |  | | 3 |  | |  | |  |  |  |
| 13 | Supramarginal gyrus | 2/40 | | R | | 10 | 10 | | 52 | | -30 | 36 | 3.34 |
|  | *SK > NK in the “suppressing negative emotion” vs. “looking at negative pictures” contrast* | | | | | | | | | | | | |
| 1 | Temporal pole | 38 | | R | | 18 | 20 | | 56 | | 6 | -6 | 4.22 |
|  | Superior Temporal gyrus |  | |  | | 2 |  | |  | |  |  |  |
| 2 | Post central gyrus |  | | R | | 5 | 10 | | 48 | | -12 | 22 | 3.8 |
|  | Superior parietal gyrus |  | |  | | 5 |  | |  | |  |  |  |
| 3 | Insula | 13 | | L | | 14 | 14 | | -32 | | -26 | 18 | 3.61 |

Note. BA=Brodmann Area, H=Hemisphere, NK=North Korean refugees, SK=South Korean controls


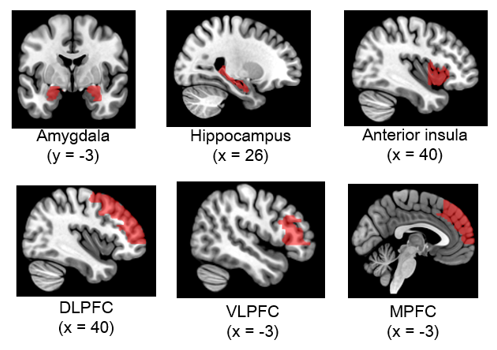


**Figure S1.** Anatomically-defined regions of interest (ROIs) based on the automated anatomical labeling (AAL). Note. DLPFC = dorsolateral prefrontal cortex, VLPFC = ventrolateral prefrontal cortex, MPFC = medial prefrontal cortex


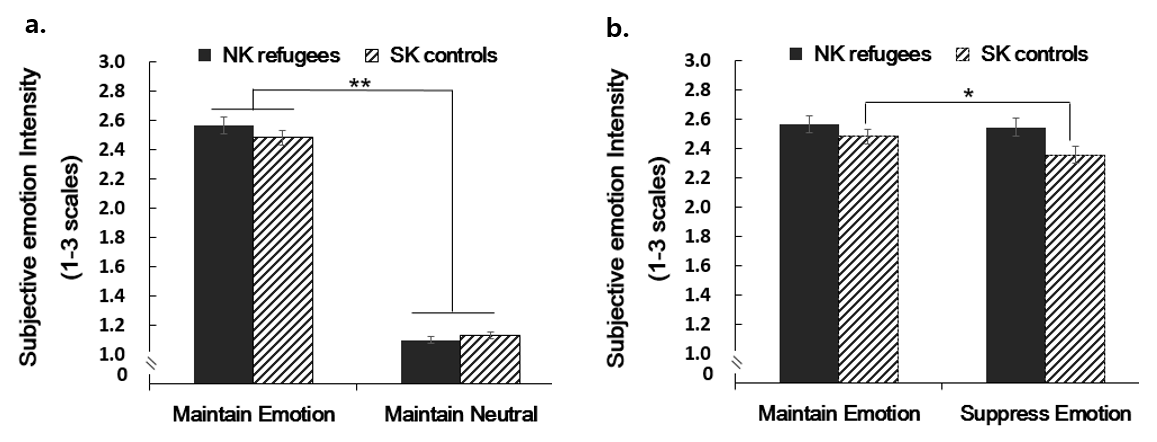


**Figure S2.** Behavioral ratings after the emotion regulation trials. a) The significant main effect of the condition: both SK controls and NK refugees reported more intense responses after maintaining responses to negative pictures compared to neural pictures. b) The significant group X condition interaction effect: SK controls reported less intense emotional responses after suppressing responses to negative pictures compared to after maintaining responses to negative pictures, but NK refugees did not show such reduction in emotional ratings.


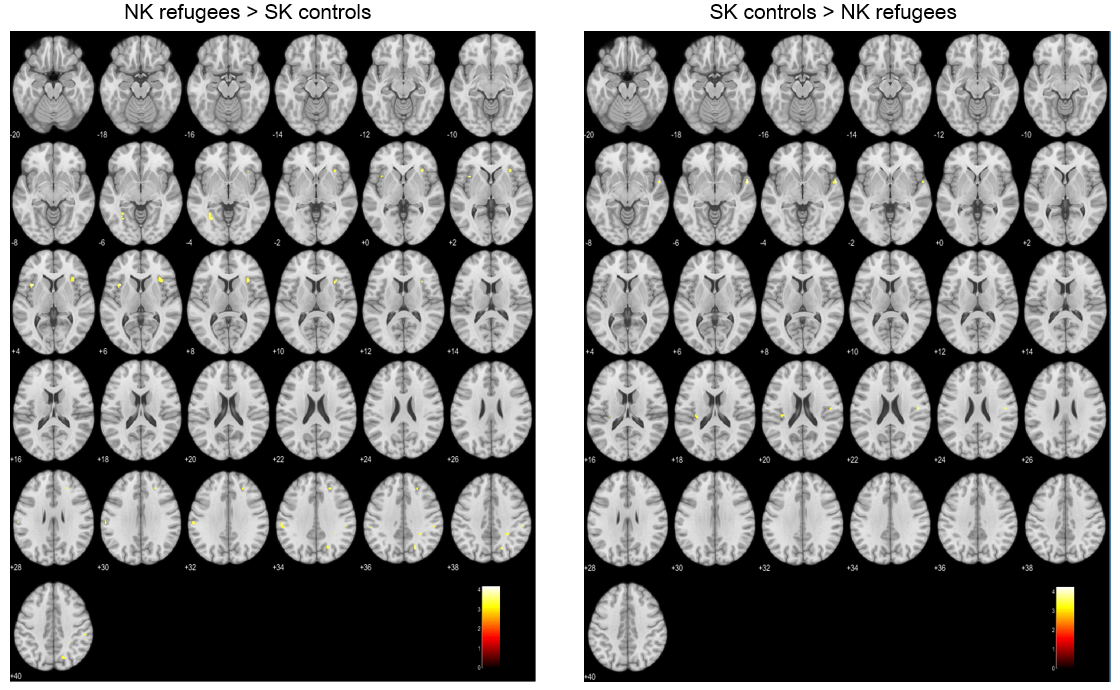


**Figure S3.** NK refugees > SK controls (left) and SK controls > NK refugees (right). Brain regions showing group differences during emotion suppression (vs. looking at negative pictures)


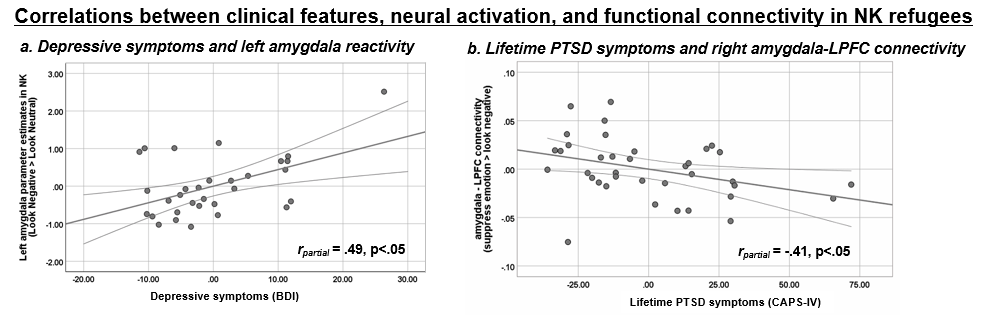


**Figure S4**. Partial correlations between clinical features, neural activation (emotion reactivity) and functional connectivity (emotion regulation) within NK refugees, controlling for age, gender, the number of traumas, and alexithymia: a. Partial correlation between depressive symptoms (BDI) and left amygdala reactivity in response to negative socio-affective pictures (vs. neutral pictures), b. Partial correlation between lifetime PTSD symptoms and right amygdala-left lateral prefrontal cortex (LPFC) during emotion suppression (vs. looking at negative pictures). These results did not remain significant after correction for multiple correlation tests using the Benjamini-Hochberg method with a false discovery rate (FDR) of 0.05.

Note. NK = North Korean, BDI=Beck Depression Inventory, PTSD = Post-Traumatic Stress Disorder, CAPS-IV = Clinician-Administered PTSD Scale-IV.

**References**

1 Miller, A. B. *et al.* Neural Correlates of Emotion Regulation and Adolescent Suicidal Ideation. *Biol Psychiatry Cogn Neurosci Neuroimaging* **3**, 125-132, doi:10.1016/j.bpsc.2017.08.008 (2018).

2 Cox, R. W. AFNI: software for analysis and visualization of functional magnetic resonance neuroimages. *Comput Biomed Res* **29**, 162-173, doi:10.1006/cbmr.1996.0014 (1996).

3 McLaren, D. G., Ries, M. L., Xu, G. & Johnson, S. C. A generalized form of context-dependent psychophysiological interactions (gPPI): a comparison to standard approaches. *Neuroimage* **61**, 1277-1286, doi:10.1016/j.neuroimage.2012.03.068 (2012).

4 Whitfield-Gabrieli, S. & Nieto-Castanon, A. Conn: a functional connectivity toolbox for correlated and anticorrelated brain networks. *Brain Connect* **2**, 125-141, doi:10.1089/brain.2012.0073 (2012).

5 Desikan, R. S. *et al.* An automated labeling system for subdividing the human cerebral cortex on MRI scans into gyral based regions of interest. *Neuroimage* **31**, 968-980, doi:10.1016/j.neuroimage.2006.01.021 (2006).
